# Clustering Transmission Opportunity Length (CTOL) Model over Cognitive Radio Network

**DOI:** 10.3390/s18124351

**Published:** 2018-12-10

**Authors:** Mas Haslinda Mohamad, Aduwati Sali, Fazirulhisyam Hashim, Rosdiadee Nordin, Osamu Takyu

**Affiliations:** 1 Research Centre of Excellence for Wireless and Photonics Network (WiPNET), Department of Computer and Communication Systems Engineering, Faculty of Engineering, Universiti Putra Malaysia, Serdang 43400, Selangor, Malaysia; aduwati@upm.edu.my (A.S.); fazirul@upm.edu.my (F.H.); 2 Center for Telecommunication Research & Innovation (CeTRI), Fakulti Kejuruteraan Elektronik dan Kejuruteraan Komputer (FKEKK), Universiti Teknikal Malaysia Melaka (UTeM), Hang Tuah Jaya, Durian Tunggal 76100, Melaka, Malaysia; 3 Centre of Advanced Electronic & Communication Engineering, Faculty of Engineering and Built Environment, Universiti Kebangsaan Malaysia, Bangi 43600, Malaysia; adee@ukm.edu.my; 4 Faculty of Engineering Electrical and Computer Engineering, Shinshu University, 4-17-1, Wakasato, Nagano City 380 8553, Japan; takyu@shinshu-u.ac.jp

**Keywords:** cognitive radio, opportunistic access, primary user, secondary user, transmission opportunity length, WLAN

## Abstract

This paper investigated the throughput performance of a secondary user (SU) for a random primary user (PU) activity in a realistic experimental model. This paper proposed a sensing and frame duration of the SU to maximize the SU throughput under the collision probability constraint. The throughput of the SU and the probability of collisions depend on the pattern of PU activities. The pattern of PU activity was obtained and modelled from the experimental data that measure the wireless local area network (WLAN) environment. The WLAN signal has detected the transmission opportunity length (TOL) which was analyzed and clustered into large and small durations in the CTOL model. The performance of the SU is then analyzed and compared with static and dynamic PU models. The results showed that the SU throughput in the CTOL model was higher than the static and dynamic models by almost 45% and 12.2% respectively. Furthermore, the probability of collisions in the network and the SU throughput were influenced by the value of the minimum contention window and the maximum back-off stage. The simulation results revealed that the higher contention window had worsened the SU throughput even though the channel has a higher number of TOLs.

## 1. Introduction

Dynamic spectrum access (DSA) is one of the cognitive radio (CR) technologies, and it is used to utilise the spectrum proficiently. The purpose of DSA is to use the primary user (PU) channel that is sparsely occupied by other temporary users such as the secondary users (SU). Opportunistic spectrum access (OSA) or commonly known as the spectrum overlay is one of the DSA schemes which has the best compatibility with the static PU transmission [1]. Accordingly, the OSA enables the SU to access a channel when the PU is detected as an idle state through the spectrum sensing process (SSP). Previous studies [2,3,4] have reported that the longer sensing duration has reduced not only the collision probability but also introduced a time overhead that decreases the SU throughput. Hence, this study intended to investigate the compromise between the increment of SU throughput and the reduction of the interference to understand the fundamental performance of the CR network.

Spectrum opportunities or also known as the transmission opportunity length (TOL) can be detected using spectrum sensing and may influence the SU performance. In [2] the length of contention phase in small-scale-backoff-based of MAC protocol (SMAC) has been studied. The SU will not interfere during the contention phase but only can use the remaining period to transmit data. The remaining period is considered to be TOL for SU to transmit data. The longer TOL may increase more chances of the SU to access a channel compared to the smaller TOL. According to [2,4], TOL durations provide a variable impact in the performance of SU throughput and interference to the PU. Therefore, the strategy that is used to access the channel should consider analyzing the length of TOL from the PU activity patterns to obtain a better SU performance.

The behavior of the SU networks is influenced by the spectrum occupancy patterns in PU networks [5]. Hence, the accuracy of the PU activity model is considered to be an important factor. The PU activity durations are usually modelled as an exponential distribution of random variables in [6,7,8,9]. According to several empirical measurement studies [10,11,12,13] on the time duration of PU activity, it is not exponentially distributed in the actual system [6]. Moreover, the prior spectrum usage models that have been widely used are based on several assumptions which have not been validated by empirical evidence [14,15].

In this study, the PU activity is obtained from the real-time experimental or measurement data. This study has used an experimental setup for a wireless local area network (WLAN) to measure the TOL in the system in which WLAN is emulated as a PU to represent the random PU activity. Furthermore, an empirical model based on the primary user traffic for opportunistic access (EM-PuO) is introduced as an empirical model of the PU channel usage pattern. The model offers access to the SU based on a realistic wireless environment, where the PU activity pattern is modelled based on an actual WLAN environment. Besides that, energy detection is used to detect the PU and extract the TOL. The TOL is clustered into two categories which are small and large, and the first order of the Markov model is used to obtain the PU activity pattern based on the clustering of the TOL. The main contributions of this paper are summarized as follows:

1. EM-PuO model: The PU activity traffic pattern model is designed based on a realistic (i.e., real-time) wireless environment which is WLAN. The EM-PuO is an empirical model of the measured WLAN signal. This model presents a temporal characterization of the detection TOL which approaching the real situation as it was constructed by the experiment in a wireless environment. This empirical model will be used to demonstrate the SU access strategy in the CTOL model.
2. Clustering TOL model: A new SU access strategy from clustering the durations of TOL. The TOLs in the EM-PuO model was analyzed and then classified into two types of TOL which are large and small. The CTOL model is designed using a Markov model that formulated the two states using the clustered TOLs. The probability of collision and the SU throughput were investigated using CTOL model by considering the dynamic PU traffic.

## 2. Related Work

It was known that several spectrum access techniques used mathematical models such as Markov decision process (MDP) [16], queuing theoretic [17], and game theoretic [18]. A multiple access strategy with cooperative relays was proposed in [17,19,20] where the SUs were modelled as a separate queuing system. A coalitional game theoretic approach is presented in [18,21], while Jiang et al. in [22] proposed a joint spectrum sensing and access framework that used evolutionary game theory. The other spectrum or channel access technique is using rendezvous scheme which does not rely on the common control channel (CCC) to control messages before starting data transmission. According to [23,24] this approach can overcome the dense in the CR network.

There are two types of PU activity models that are used as references: (1) the first model is a static PU model, which is a detection model, and it assumes that the PU is either present or absent during the SU frame duration [4,25]; and (2) the second model is known as the dynamic PU model, which is a cross-layer approach indicating the PU’s traffic model based on its arrival and departure time [2,4,26]. The dynamic PU model is designed to demonstrate a realistic situation where the PU may randomly arrive or depart from the channel at any time.

The trade-off between the sensing and SU throughput was investigated to get a better quality of sensing time without degrading the achievable throughput. The quality of sensing can be improved by increasing the sensing period, but it will affect the SU throughput as a result of fixing the overall frame duration. The sensing-throughput trade-off was studied in [27,28] by assuming that the PU is static, while the dynamic PU traffic was addressed in [2,4,26].

## 3. System Model

**Figure 1:**
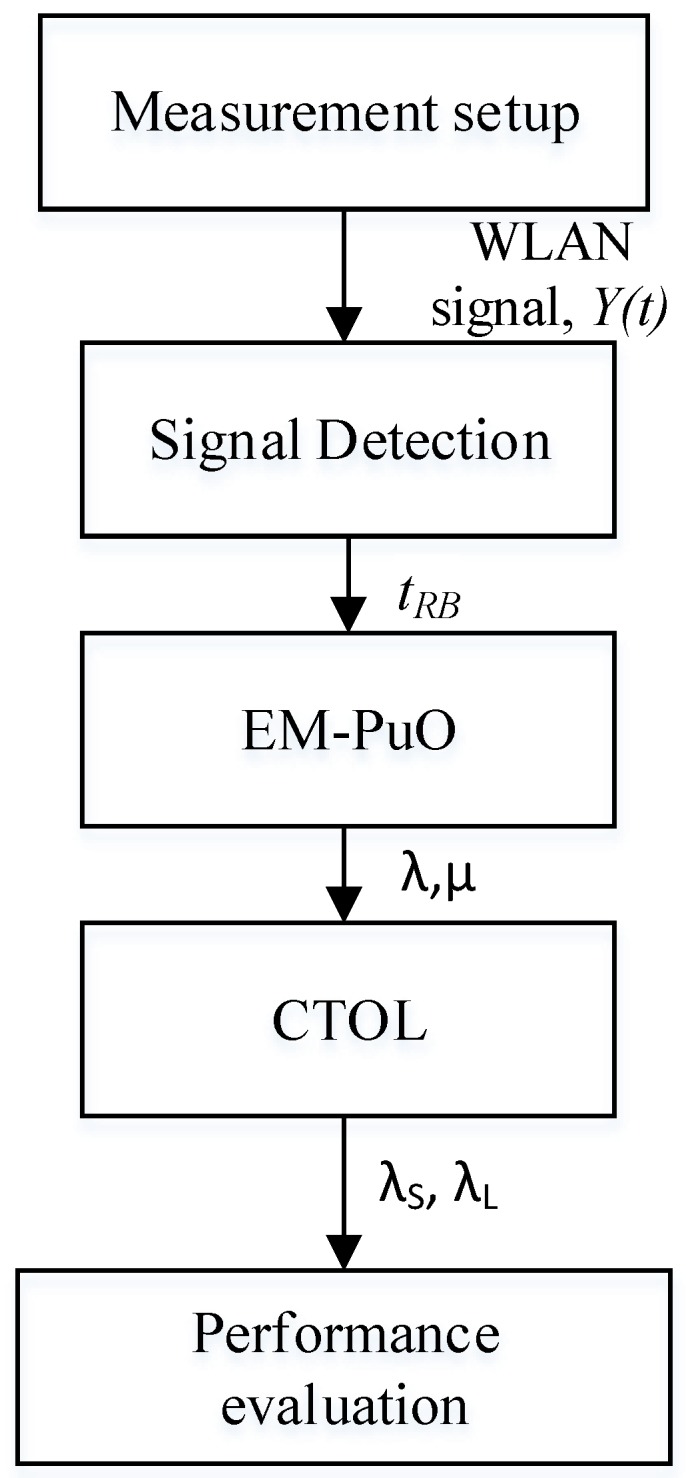
Flow chart of the CTOL system.

The system model is an ad hoc network consisting of a pair of primary transmitters, and the primary destination shares a channel with a pair of SUs. The SUs communicate with each other using direct transmission by identifying the spectrum holes via spectrum sensing. The flow of the CTOL system can be categorized into five main parts. The parts are included the measurement of WLAN signal and detection, modelled the EM-PuO, and clustered the TOL and performance evaluation. Figure 1 shows the sequence of the parts.

**Figure 2:**
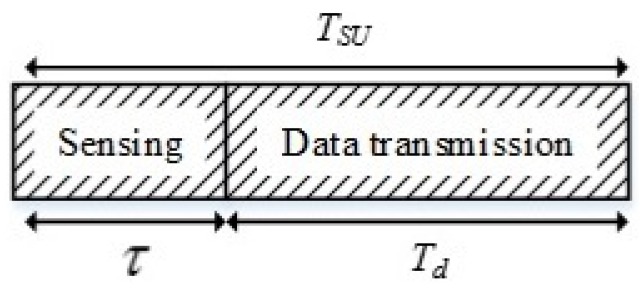
Secondary user (SU) frame structure.

The SU frame structure is divided into two segments which are sensing time, τ and data transmission time, $\;T_{d}$, as shown in Figure 2. The frame duration is equal to $\;T_{SU}=T_{d}+\tau$, and the total number of samples in a frame is denoted as J. It is assumed that the SU operation occurs within one frame and the energy detection is performed during the sensing period. According to the Nyquist theorem, the sampling interval is set as $\;T_{s}=\frac{1}{B}$, where B is the channel bandwidth and I=Bτ is the total number of samples.

### 3.1. Measurement Setup

**Figure 3:**
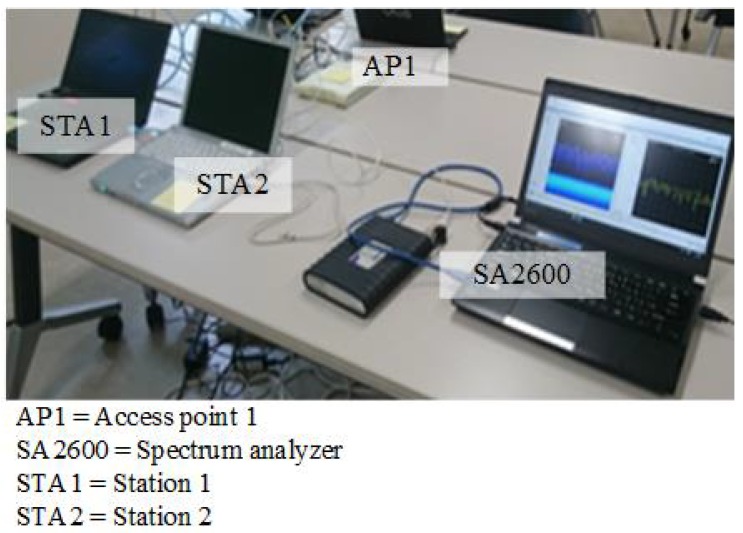
The experimental setup of the WLAN networks.

An experimental setup measures the WLAN signal to demonstrate a random PU activity that resembles a real-time wireless environment. There are two stations (STAs) and an access point (AP1) in the experiment. The two STAs are identified as STA1 and STA2, which are connected to AP1 through a wired and wireless LAN, respectively. Both STA1 and STA2 share a significant amount of the data (i.e., file) through AP1. STA2 retrieves the data file from STA1 using the MS Windows file sharing facility. The large data file is used to avoid the download process from being completed during the measurement process. Therefore, the resultant traffic via the access to the WLAN is considered to be full-buffering. Table 1 shows the specification of the WLAN system in the experiment. The detecting antenna (DA) has discovered a packet in the system, which is a wireless LAN Omni-antenna. The DA is connected to a real-time spectrum analyzer, SA2600 (Techtronic, SW Karl Braun Drive, OR, USA), to display the spectral activities of the system in an indoor real-time environment. The measurement antenna is located near STA1 and AP1 to maintain the power of the signal. As a result, the setup can avoid false alarms and misdetections. Figure 3 shows the structure of the experimental setup.

### 3.2. The Detection of the WLAN (802.11) Signal

The detected signal of the wireless network is observed from AP1 and displayed in the SA2600 spectrum analyzer, which is then saved for offline processing and analysis. The displayed signal is emulated as PU activity in a channel, and the parameters of the signal detector are listed in Table 2.

The active and idle states of the channel are extracted from the experimental data based on the energy detection which is the preferred approach in many prior studies due to its simplicity and relevance in processing the measurement power [14,29,30]. The energy detection compares the received signal energy in a certain channel to a correctly established decision threshold. The hypotheses model of the detection technique is defined as:

(1) $$\begin{array}{c} \begin{array}{c} Y=\left\{\begin{array}{cc} \sum_{i=1}^{I}n_{i}^{2}, & H_{0}\\ \;\sum_{i=1}^{I}s_{i}^{2}+n_{i}^{2}, & H_{1} \end{array}\right. \end{array} \end{array}$$

where $\;n_{i}$, with i∈[1,2…I] is the sample of Gaussian white noise, and $\;s_{i}$, with i∈[1,2…I] are the samples of the PU signals. The $\;H_{0}$ indicates that the spectrum bands are detected as being idle, and $\;H_{1}$ indicates that the PU occupies a channel.

The SU data transmission is activated based on the spectrum sensing result, where the SU will be transmitted during $\;H_{0}$ while remaining silent during $\;H_{1}$. The threshold for the targeted $\;P_{d}$ and $\;P_{f}$ is formulated as [31]:

(2) $$\lambda_{P_{d}}=\sigma_{n}^{2}\left(\sqrt{\frac{2(2\gamma +1)}{M}}Q^{-1}\left(\bar{P_{d}}\right)+\gamma +1\right)$$

(3) $$\lambda_{P_{f}}=\sigma_{n}^{2}\left(\sqrt{\frac{2}{M}}Q^{-1}\left(\bar{P_{f}}\right)+\gamma +1\right)$$

where $\;P_{d}$ and $\;P_{f}$ are the target detection probability and false alarm probability, whereas $\;\sigma_{n}^{2}$ is the noise variance, γ is the signal to noise ratio, and M is the number of samples.

**Figure 4:**
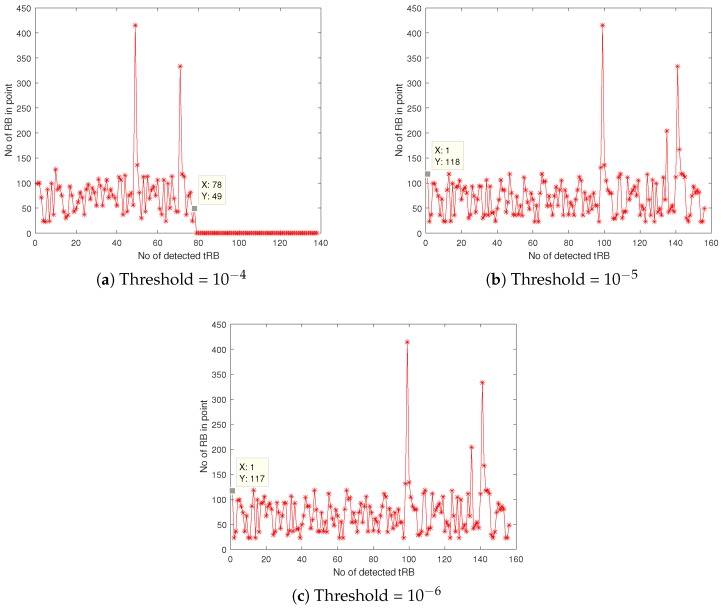
The number of idle times detected $\;(t_{RB})$ with different threshold values: (**a**) $\;10^{-4}$, (**b**) $\;10^{-5}$ and (**c**) $\;10^{-6}$.

Figure 4 illustrates the number of idle period ($t_{RB}$) detected with three different thresholds which are $\;10^{-4}$, $\;10^{-5}$ and $\;10^{-6}$. In Figure 4a the number of detected $\;t_{RB}$ is 78, which is the lowest among the other threshold decisions. For Figure 4b,c, the number of detected $\;t_{RB}$ is the same which is equal to 156. The received signal is then compared with the correctly established threshold value, which is $\;10^{-5}$. The threshold value is chosen since it allows the received signal sense higher numbers of $\;t_{RB}$ compared to the threshold value of $\;10^{-4}$, in addition to allow detection of longer idle times, compared to the threshold of $\;10^{-6}$.

## 4. Primary User Traffic Model Based on the Empirical Model: EM-PuO Design

The PU activity model is based on a real-time experimental data. An experimental setup to measure the TOL of a WLAN is employed in Section 3.1. The extracted TOL from the WLAN signal is analyzed and modelled as EM-PuO. The EM-PuO is the empirical measurement data of the PU, which is emulated by the WLAN system as random PU activity for SU opportunistic transmission. The modelled EM-PuO is used to determine the realistic spectrum occupancy of the PU channels based on the actual measurement of the WLAN system.

### 4.1. Analysis of the Detected WLAN (802.11) Signal

**Figure 5:**
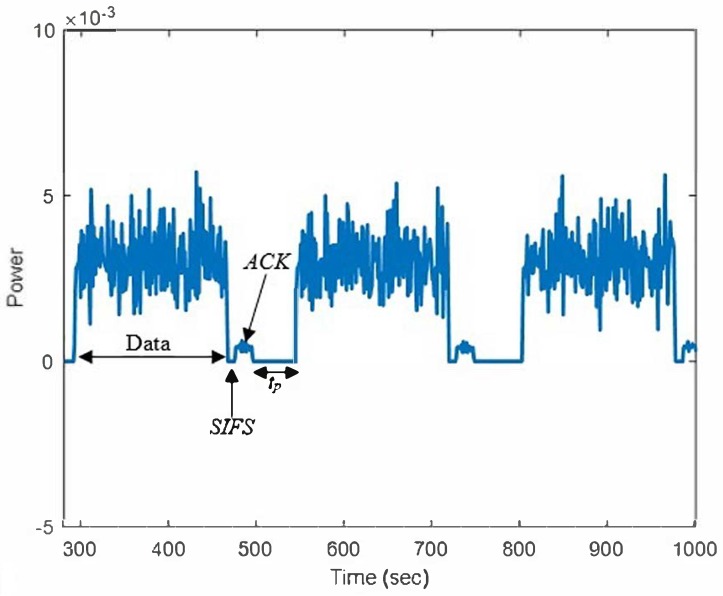
The analysed WLAN signal using the spectrum analyzer.

The obtained signal is analyzed according to the MAC protocol of the IEEE 802.11a standard. The signal includes the data packet, short inter-frame spacing (SIFS), ACK, and stop period, $\;t_{p}$. The $\;t_{p}$ is a space that consists of distributed coordination function spacing (DIFS), and the random back-off time ($t_{RB}$). Figure 5 shows the analyzed WLAN signal. In the standard transmission data packet, the appearance of SIFS indicates the end of the data packet, and the ACK signal is sent by AP1 to acknowledge the received packet.

The appearances of the data series and SIFS, ACK, and DIFS spaces thereby indicate that the channel is busy and is known as $\;t_{busy}$. While running the random back-off time, the channel is identified as $\;t_{idle}$ (PU ‘off’state). The states of $\;t_{busy}$ and $\;t_{idle}$ are expressed as given by the following equations:

(4) $$t_{busy}=t_{DATA}+t_{SIFS}+t_{ACK}+t_{p}$$

(5) $$\begin{array}{c} \begin{array}{c} t_{RB}=t_{p}-t_{DIFS} \end{array} \end{array}$$

(6) $$\begin{array}{c} \begin{array}{c} t_{idle}=t_{RB} \end{array} \end{array}$$

where $\;t_{RB}$ is time for random back-off.

In this system, the PU also performs sensing to detect the wireless access. If there is any access detected during $t_{RB}$, the countdown of the back-off is immediately stopped. During measurement, the SU transmitter and receiver are configured to communicate through a short-range communication with minimum signal power. Even though the SU signal is low, it still produces harmful interference to PU due to the close distance between the SU transmitter and PU receiver. In this situation, the PU could not detect any access in the system but suffers from the hidden node terminal interference. In order to avoid this problem, the SU is only used to exploit the spectrum during the back-off period, $t_{RB}$.

### 4.2. The Probability Distribution of Idle Time

Based on the extracted length of $\;t_{idle}$ and $\;t_{busy}$ obtained from the empirical data, a cumulative distribution function (CDF) was derived and compared to the probability of the distribution model. Notably, the exponential distribution is used to fit with the obtained empirical curves to present the duration of the idle and busy states in the statistical properties. Assuming that the exponential matches or corresponds to the idle periods, it can be defined as follows:

(7) $$\begin{array}{c} \begin{array}{c} F_{T_{idle}}=1-e^{-\lambda t_{idle}} \end{array} \end{array}$$

where the estimated λ of the considered exponential distribution that uses the relation is as follows:

(8) $$\begin{array}{c} \begin{array}{c} \lambda =\frac{1}{E[T_{idle}]} \end{array} \end{array}$$

Next, the Kolgomorov-Sminorv (KS) test is calculated as [32] for both empirical data and exponential fit to quantify the distance, $\;D_{KS}$:

(9) $$\begin{array}{c} \begin{array}{c} D_{KS}=\max_{T_{idle}}\left\{\right|F_{T_{idle}}^{e}\left(t_{idle}\right)-F_{T_{idle}}\left(t_{idle}\right)\left|\right\} \end{array} \end{array}$$

where $\;F_{T_{idle}}$ is empirical cdf of $\;T_{idle}$. After running the KS test, the $\;t_{idle}$ and $\;t_{busy}$ are approximately the exponential distribution random variables as λ and μ respectively.

## 5. Clustering Transmission Opportunity Length (CTOL) Model

The CTOL model represents the presence and absence of PU, which is similar to [2] which is based on the PU arrival and departure time. The channel occupancy contains of two states, P={0,1} in which represent the channel are idle and occupied, respectively. In this model, the TOLs are clustered or separated into two groups that are denoted as large TOL, $\;t_{idleL}$ and small TOL, $\;t_{idleS}$. The $\;t_{idleL}$ is defined as idle states, while $\;t_{idleS}$ is assumed as busy in P.

There are two states of SU frame structure, S={0,1} in which 0 represents the sensing time state while and 1 is the data transmission time. No collision will happen when the SU state is 0 and collision may occur during 1. Hence, there are four different states of PU’s traffic in the CTOL model, which are based on the channel occupancy and SU frames structure during the transmission. The four states are: (i) $\;H_{00}^{N}\left(x\right)$; (ii) $\;H_{01}^{N}\left(x\right)$; (iii) $\;H_{10}^{N}\left(x\right)$; and (iv) $\;H_{11}^{N}\left(x\right)$.

### 5.1. CTOL Protocol Design

The duration of $\;t_{idle}$ is a critical factor that enables the SU to access the channel. Therefore, it is important to analyze $\;t_{idle}$ so that the throughput of the SU is enhanced without causing any harmful interferences to the PU. In the CTOL model, the detected $\;t_{idle}$ undergoes the clustering process that is detached to the large and small idle time. The transmission opportunity length of the channel is defined as follows:

(10) $$\begin{array}{c} \begin{array}{c} TOL=\left\{\begin{array}{cc} t_{idleS}, & t_{idle}\leq t_{th}\\ \;t_{idleL}, & t_{idle}>t_{th} \end{array}\right. \end{array} \end{array}$$

where $\;t_{idleL}$ and $\;t_{idleS}$ are large and small idle times, respectively and $t_{th}$ is the threshold values of the random back-off time. The $\;t_{th}$ is determined by considering the range of the total $t_{idle}$ in the system and the contention window (*W*) value.

From the classification of $\;t_{idle}$, the opportunity length of the time required to access a channel is modelled using the two-state Markov model. The PU activity is modelled as Q based on the assumption that $\;t_{idleL}$ offers an idle channel for the access of SU. Meanwhile, $\;t_{idleS}$ is assumed not suitable for the SU transmission because it will be exposed to the interference in the channel. The mean values of $\;t_{idleS}$ and $t_{idleL}$ in the exponential distribution are given as $\;\lambda_{S}$ and $\;\lambda_{L}$, respectively.

Additionally, the probability of the transition of $\;t_{idleL}$ time from large to large and small to large are formulated as follows:

(11) $$\begin{array}{c} \begin{array}{c} q_{00}=\frac{N_{LL}}{N_{L}} \end{array} \end{array}$$

(12) $$\begin{array}{c} \begin{array}{c} q_{01}=\frac{N_{LS}}{N_{L}} \end{array} \end{array}$$

(13) $$\begin{array}{c} \begin{array}{c} q_{10}=\frac{N_{SL}}{N_{S}} \end{array} \end{array}$$

(14) $$\begin{array}{c} \begin{array}{c} q_{11}=\frac{N_{SS}}{N_{S}} \end{array} \end{array}$$

where $\;N_{L}$ is the total number of $\;t_{idleL}$ and $\;N_{S}$ is the total number of $\;t_{idleS}$. Meanwhile $\;N_{LL}$ is the number of TOL transition from $\;t_{idleL}$ to $\;t_{idleL}$, $\;N_{LS}$ is the transition from $\;t_{idleL}$ to $\;t_{idleS}$, $\;N_{SL}$ is transition from $\;t_{idleS}$ to $\;t_{idleL}$, and $\;N_{SS}$ is the transition from $\;t_{idleS}$ to $\;t_{idleS}$.

### 5.2. PU Traffic Model

**Figure 6:**
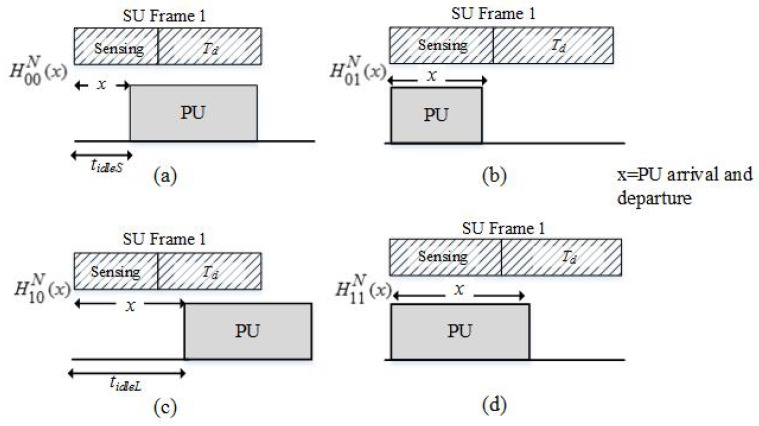
The PU’s traffic model of the CTOL model.

A realistic PU traffic model is then developed as in [2] to analyze the detection of the PU’s signal by the SUs during spectrum sensing. The detected WLAN signal in Section 3.2 is emulated as the PU traffic pattern. The CTOL model uses the empirical data from the measured WLAN signal and analyzed them randomly. This model uses equations in [2] as references to develop the random arrival and departure times of PU. Figure 6 shows the PU’s random departure and arrival times within one frame of SU that considers the duration of $t_{idleS}$ and $\;t_{idleL}$.

The different scenarios of PU traffic pattern in CTOL model are indicated using the notation of $\;H_{ps}^{N}\left(x\right)$. The subscript p indicates the channel occupancy status, s is the SU frame structure states, and x represents the PU arrival and departure time. The superscript N represents the new model, which is the CTOL model.

Initially, in $\;H_{00}^{N}\left(x\right)$ and $\;H_{01}^{N}\left(x\right)$, the PU is absent, and the noise is only detected during the sensing period. However, the PU arrives during the xth sample of the sensing period in $\;H_{00}^{N}\left(x\right)$, and the PU signal is then detected. Meanwhile, in $\;H_{10}^{N}\left(x\right)$ and $\;H_{11}^{N}\left(x\right)$, the channel is detected as busy at the beginning of SU transmission. In $\;H_{10}^{N}\left(x\right)$, the PU departs from the channel during the sensing period, while $\;H_{11}^{N}\left(x\right)$ is the hypothesis that the PU is always present during the sensing period. The transition probability of the CTOL model is given by [33]:

(15) $$\begin{array}{c} \begin{array}{c} Q_{\phi \theta }^{N}=\left[\begin{array}{ll} q_{00}\left(T_{s}\right) & q_{01}\left(T_{s}\right)\\ q_{10}\left(T_{s}\right) & q_{11}\left(T_{s}\right) \end{array}\right] \end{array} \end{array}$$

where $\;q_{00}$, $\;q_{01}$, $\;q_{10}$, and $\;q_{11}$ are the probabilities of transition between large and small states and vice-versa.

The probabilities of the hypotheses for the CTOL model were formulated from [2,4,26] as shown in Figure 5. The Equations of (16)–(19) are reformulated from [2] which incorporates the clustered TOL; $\;t_{idleS}$ and $\;t_{idleL}$ from Equation (10). The probabilities of the hypotheses are considered as the probability of small and large idle time which can be derived as follows:

(16) $$\begin{array}{c} \begin{array}{c} p_{H_{00}^{N}}\left(x\right)=p_{S}q_{00}^{x}\left(T_{s}\right)q_{01}\left(T_{s}\right)q_{00}^{J-x-1}\left(T_{s}\right),\;I\leq x\leq I-1 \end{array} \end{array}$$

(17) $$\begin{array}{c} \begin{array}{c} p_{H_{01}^{N}}\left(x\right)=\left\{\begin{array}{ll} p_{L}q_{00}^{x}\left(T_{s}\right)q_{01}\left(T_{s}\right)q_{00}^{J-x-1}\left(T_{s}\right), & I\leq x\leq J\\ \;p_{L}q_{00}^{J}\left(T_{s}\right), & x=J \end{array}\right. \end{array} \end{array}$$

(18) $$\begin{array}{c} \begin{array}{c} p_{H_{10}^{N}}\left(x\right)=p_{b}q_{11}^{x}\left(T_{s}\right)q_{10}\left(T_{s}\right)q_{00}^{J-x-1}\left(T_{s}\right),\;I\leq x\leq J \end{array} \end{array}$$

(19) $$\begin{array}{c} \begin{array}{c} p_{H_{11}^{N}}\left(x\right)=\left\{\begin{array}{ll} p_{b}q_{00}^{x}\left(T_{s}\right)q_{01}\left(T_{s}\right)q_{00}^{J-x-1}\left(T_{s}\right), & I\leq x\leq J\\ \;p_{b}q_{11}^{J}\left(T_{s}\right), & x=J \end{array}\right. \end{array} \end{array}$$

where $\;p_{S}=\frac{\lambda_{S}}{\lambda_{S}+\lambda_{L}}$ and $\;p_{L}=\frac{\lambda_{L}}{\lambda_{S}+\lambda_{L}}$ represent the probability of $\;t_{idleS}$ and $\;t_{idleL}$, respectively. The SU transmission rate is derived based on the hypotheses for the improved throughput [4] and can be expressed as:

(20) $$\begin{array}{c} \begin{array}{c} r_{H_{i}\left(x\right)}=B.\log_{2}\left(1+\frac{\gamma_{SU}}{1+\gamma_{H_{i(x)}}}\right) \end{array} \end{array}$$

The $\;\gamma_{H_{00}\left(x\right)}=\frac{\left(I-x\right)\gamma_{PU}}{I}$, $\gamma_{H_{01(x)}}=0$, $\;\gamma_{H_{10}\left(x\right)}=\frac{\left(x\right)\gamma_{PU}}{I}$ and $\gamma_{H_{11(x)}}=\gamma_{PU}$ are the average SNRs that considered the random arrival and departure of PU traffic.

### 5.3. The Probability of Collision

The probability of the collision between the SU and the PU is observed through imperfect sensing. In a real-time environment, the sensing errors can occur in spectrum sensing. The probability of the collision between SU and PU is denoted as $\;p_{coll}^{1}$. The spectrum sensing results might have errors that can lead to false alarms and missed detection.

This study assumed that the sensing errors such as the probability of false alarms could occur in a channel. The SU frames might collide with the PU’s transmissions if the channel is idle with the presence of PU’s transmissions during the transmission period.

From Figure 6, the conditional probability of collisions of the CTOL model can be calculated as follows:

(21) $$\begin{array}{c} \begin{array}{c} p_{coll}^{1}=\sum_{x=1}^{I-1}p_{H_{00}^{N}\left(x\right)}p_{fa}+\sum_{x=1}^{I-1}p_{H_{01}^{N}\left(x\right)}p_{fa}+\sum_{x=1}^{I-1}p_{H_{11}^{N}\left(x\right)}p_{fa} \end{array} \end{array}$$

where $\;p_{fa}$ is the probability of false alarms.

When the sensing period increases, the probability of detection will also increase while the probability of false alarm decreases, and it is equal to $\;p_{fa}=1-p_{d}$.

The probability of the collision among the SUs is denoted as $\;p_{coll}^{2}$, which can occur if there is at least one SU that is transmitted in the same frame. The conditional collision probability between $\;p_{coll}^{1}$ and $\;p_{coll}^{2}$ means that the probability for the collision of the SU packet can be seen during its transmission in a channel. The conditional probability of the collision between the SUs is given as $\;p_{coll}^{2}=1-\left(1-\tau_{st}^{\left(n-1\right)}\right)$, where $\tau_{st}$ is the stable transmission probability at the beginning of the idle time.

The total conditional probability collision from both the SU and the PU to a packet can be derived as follows:

(22) $$\begin{array}{c} \begin{array}{c} p_{C}^{N}=p_{coll}^{1}+p_{coll}^{2}-p_{coll}^{1}.p_{coll}^{2} \end{array} \end{array}$$

The stable transmission probability is formulated from [34] as:

(23) $$\begin{array}{c} \begin{array}{c} \tau_{st}=\frac{2(1-2p_{C}^{N})}{2\left(1-2p_{C}^{N}\right)\left(W+1\right)+p_{C}^{N}W\left(1-{\left(2p_{C}^{N}\right)}^{M}\right)} \end{array} \end{array}$$

where W and M represent the minimum contention window value and the maximum back-off stage, respectively. Next, let the probability of at least, an SU transmitting during the transmission be $\;p_{tx}^{N}=1-{\left(1-\tau_{st}\right)}^{n}$ and the probability of the interference to a PU can be derived as in [2] as:

(24) $$\begin{array}{c} \begin{array}{c} p_{in}^{N}=p_{coll}^{1}\cdot p_{tx}^{N} \end{array} \end{array}$$

### 5.4. Normalized Throughput of the SU

The normalized throughput of the SU is defined as the portion of the period that the SU uses to transmit the data successfully. The throughput of the SU is calculated based on the successful SU’s transmission, which occurs when the channel is detected as idle, and the PU does not reappear during the transmission period.

As an example, let *n* be the number of SUs that contend in the channel, and the probability of at least one SU’s success to transmit in the channel is as follows:

(25) $$\begin{array}{c} \begin{array}{c} p_{success}^{N}=n\tau_{st}{\left(1-\tau_{st}\right)}^{n-1} \end{array} \end{array}$$

By considering the SU throughput, it is formulated as [2]:

(26) $$\begin{array}{c} \begin{array}{c} S^{N}=\frac{T_{SU}-\tau }{T_{SU}}\left(p_{H_{01}^{N}\left(J\right)}\left(p_{fa}\right)r_{H_{01}^{N}\left(J\right)}+\sum_{x=1}^{I-1}p_{H_{10}^{N}\left(x\right)}\left(p_{fa}\right)r_{H_{10}^{N}\left(x\right)}+p_{H_{11}^{N}\left(I\right)}\left(p_{fa}\right)r_{H_{11}^{N}\left(I\right)}\right)p_{success}^{N} \end{array} \end{array}$$

where $\;T_{SU}$ is the frame duration, and τ represents the sensing duration.

## 6. Results and Discussions

In this section, the CTOL model is evaluated and compared to the static PU model and dynamic PU model. The probability of collisions and the SU’s throughput in the network are investigated for all of the models. The total frame duration is set to 30 ms, the bandwidth B is 5 MHz, and the sampling interval is Ts = 0.2 μs. The detection probability, $\;p_{d}$, is set to 80% to restrict the interference probability to below 20%.

**Figure 7:**
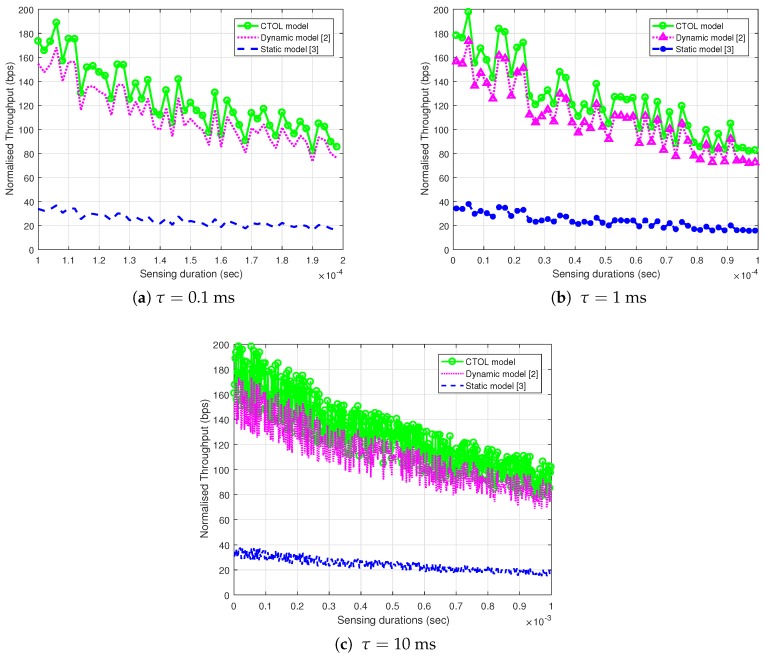
The effect of sensing the duration of the normalized throughput for the secondary user when increasing the sensing time: (**a**) τ=0.1 ms; (**b**) τ=1 ms and (**c**) τ=10 ms.

Figure 7 illustrate the relationships of the SU normalized throughput with the increment of the sensing durations for all of the models. These figures show the effect of the various sensing durations on the SU throughput. The sensing duration from 0.1 ms in Figure 7a is increased to 1 ms (Figure 7b) and 10 ms (Figure 7c). It shows that the CTOL model outperforms the other models due to the increment of the sensing duration. This happens because the static model needs a longer sensing duration to satisfy the detection constraint, in which $\;p_{d}$ is set as 0.8. The longer sensing duration improves the sensing reliability and increasing the sensing durations degrades the SU throughput. The reason for the degradation of the throughput is due to the shorter data transmission slot in the SU frames. The longer sensing duration in the frames may, shorten the data transmission time for the SU, that causes the degradation of the throughput.

The throughput performance in the CTOL model is slightly higher by 45% and 12.2% than the other two reference models. This condition occurs because the CTOL model has analyzed the TOLs and clustered them into two separate large and small parts. Then, the SU transmitted within the two TOLs to reduce the possibility of colliding with the PU as both TOLs are idle. The fluctuation of the throughput value is caused by the random values of SU transmit power in the simulation.

**Figure 8:**
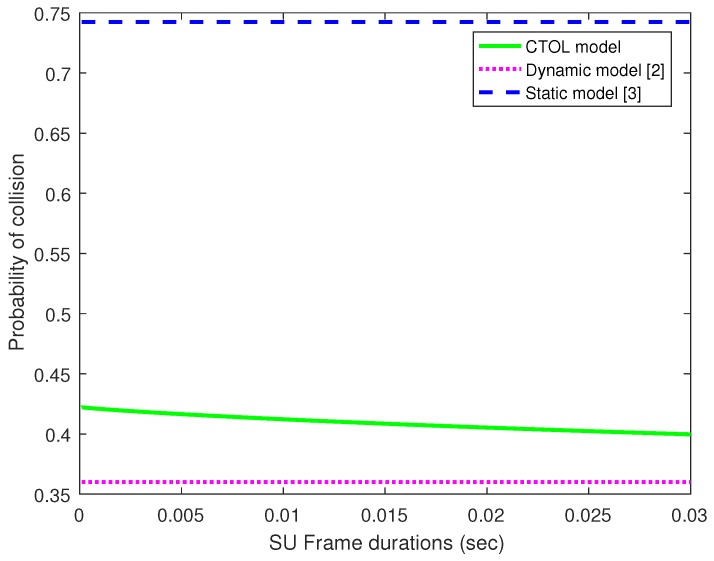
The probability of collisions for the CTOL model, dynamic model, and static model.

In Figure 8, the probability of collisions is investigated and compared to the increasing of frame duration. Then, the CTOL model is compared with the static and dynamic models. The probability of collisions in the static model shows the highest number of collisions among the other models. The static model assumed the absence and presence of the PU in the detection process. There might be the occurrence of misdetections, which will lead to a higher collision in the channel due to the imperfect sensing. Both CTOL and dynamic models use the random arrival and departure of the PU signals. These models can detect and classify the PU signals thoroughly for every sample time in the four different scenarios.

**Figure 9:**
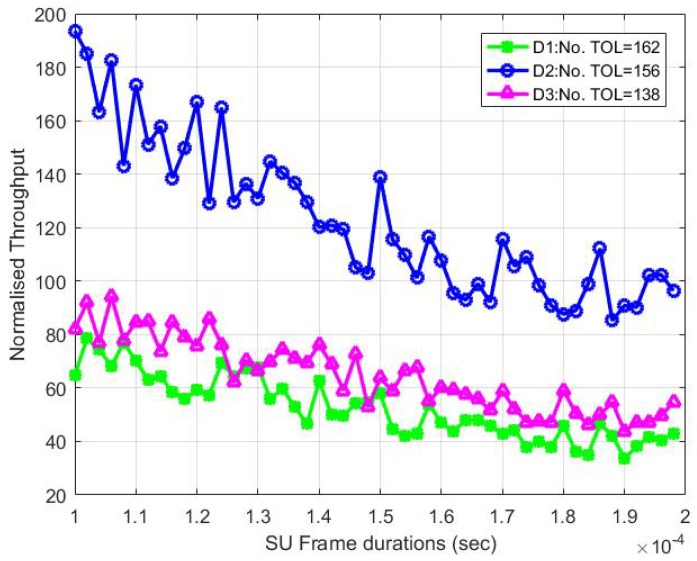
The normalized throughput for the CTOL model with the different numbers of TOL.

Figure 9 shows the SU throughput in the CTOL model for the three different transmission opportunity lengths (TOLs) in the following channels: D1, D2 and D3. The SU throughput in D2 is higher than in D1 and D3, even though it has the smallest number of TOLs compared to D1. Notably, it occurs due to the value of the probability of stable transmissions $\;\tau_{st}$, which did not only depend on the conditional probability of collisions, $\;p_{C}^{N}$, and it can also be determined by the number of W and M. Although the numbers of TOL in D1 and D3 have a large gap, the throughput is slightly different as they have the largest contention window, W=1024.

## 7. Conclusions

In this paper, the access opportunity for the SU is estimated in the channel and analyzed using an experimental model that offers an accurate and realistic scenario. From the data, the CTOL model is designed to examine the spectrum hole behaviors and evaluate all of them. The CTOL model was compared between both static and dynamic models correspondingly. The proposed CTOL model improves the SU throughput by 45% and 12.2% by clustering the detected TOLs into two large and small categories. Even though the probability of collisions in the proposed CTOL model is 13.7% higher than the dynamic model, the results showed that the performance improves as the value of the probability of collision degrades and the frame duration increases. The simulation results have proven that the SU throughput in the CTOL model can perform better with fewer minimum contention windows and maximum back-off stage.

## Figures and Tables

**Table 1:** The Specifications of the WLAN system.

WLAN Standard	IEEE 802.11a
Transmission Power	12% of the Prescribed
WLAN Extension	NEC Corp, Tokyo, Japan, PA-WL54SU2
Access Point	Logitech Corp, Tokyo, Japan, LAN-WAGE/AP

**Table 2:** Parameters of Signal detector.

Bandwidth	5 MHz
Center frequency	5.2 GHz
Reference Level	−10 dBm
Sampling rate	2 Msamples/sec
Measurement antenna	ELECOM WDC 433DU2H
Detecting Antenna	Omni-directional
